# Crystal structure of (1*S*,2*R*)-2-[(3*R*,4*S*)-3-methyl-4-phenyl-1,2,3,4-tetra­hydro­isoquinolin-2-yl]-1,2-di­phenyl­ethanol

**DOI:** 10.1107/S2056989019011964

**Published:** 2019-09-03

**Authors:** Karim Ben Ali, Pascal Retailleau

**Affiliations:** a Laboratoire de Recherche en Energie et Matière pour le Développement des Sciences Nucléaires, Centre National des Sciences et Technologies Nucléaires, Pôle Technologique, 2020 Sidi-Thabet, Tunisia; b Institut de Chimie des Substances Naturelles, CNRS UPR 2301, Université Paris-Sud, Paris-Saclay University, 1, av. de la Terrasse, 91198 Gif-sur-Yvette, France

**Keywords:** crystal structure, chiral *β*-amino alcohol, tetra­hydro­iso­quinoline, hydrogen bond, Hirshfeld surface analysis

## Abstract

The synthesis and crystal structure of the title compound, C_30_H_29_NO, are described. This compound is a member of the chiral di­hydro­iso­quinoline-derived family, used as building blocks for functional materials and as source of chirality in asymmetric synthesis, and was isolated as one of two diastereomeric *β-*amino alcohols, the title mol­ecule being found to be the (*S,R*) diastereoisomer. In the crystal, mol­ecules are packed in a herringbone manner parallel to (103) and (10) *via* weak C—H⋯O and C—H⋯π(ring) inter­actions. Hirshfeld surface analysis showed that the surface contacts are predominantly H⋯H inter­actions (*ca* 75%). The crystal studied was refined as a two-component inversion twin.

## Abstract 2

The title chiral β-amino alcohol was isolated as one of two diastereomeric *β-*amino alcohols, the title mol­ecule being found to be the (*S,R*) diastereoisomer. In the crystal, mol­ecules are packed in a herringbone manner parallel to (103) and (10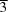) *via* weak C—H⋯O and C—H⋯π(ring) inter­actions.

## Chemical context   

*β*-amino alcohols exhibit a broad spectrum of biological activities and are used as anti­bacterial and tuberculostatic agents (Yendapally & Lee, 2008 ▸). In particular, chiral *β*-amino alcohols are very important chiral mol­ecules that are used as building blocks and structural motifs in pharmaceutically active mol­ecules and natural products and which serve as the main sources of chirality in asymmetric synthesis (Lee *et al.*, 2003 ▸; Malkov *et al.*, 2007 ▸; Guo *et al.*, 2017 ▸).
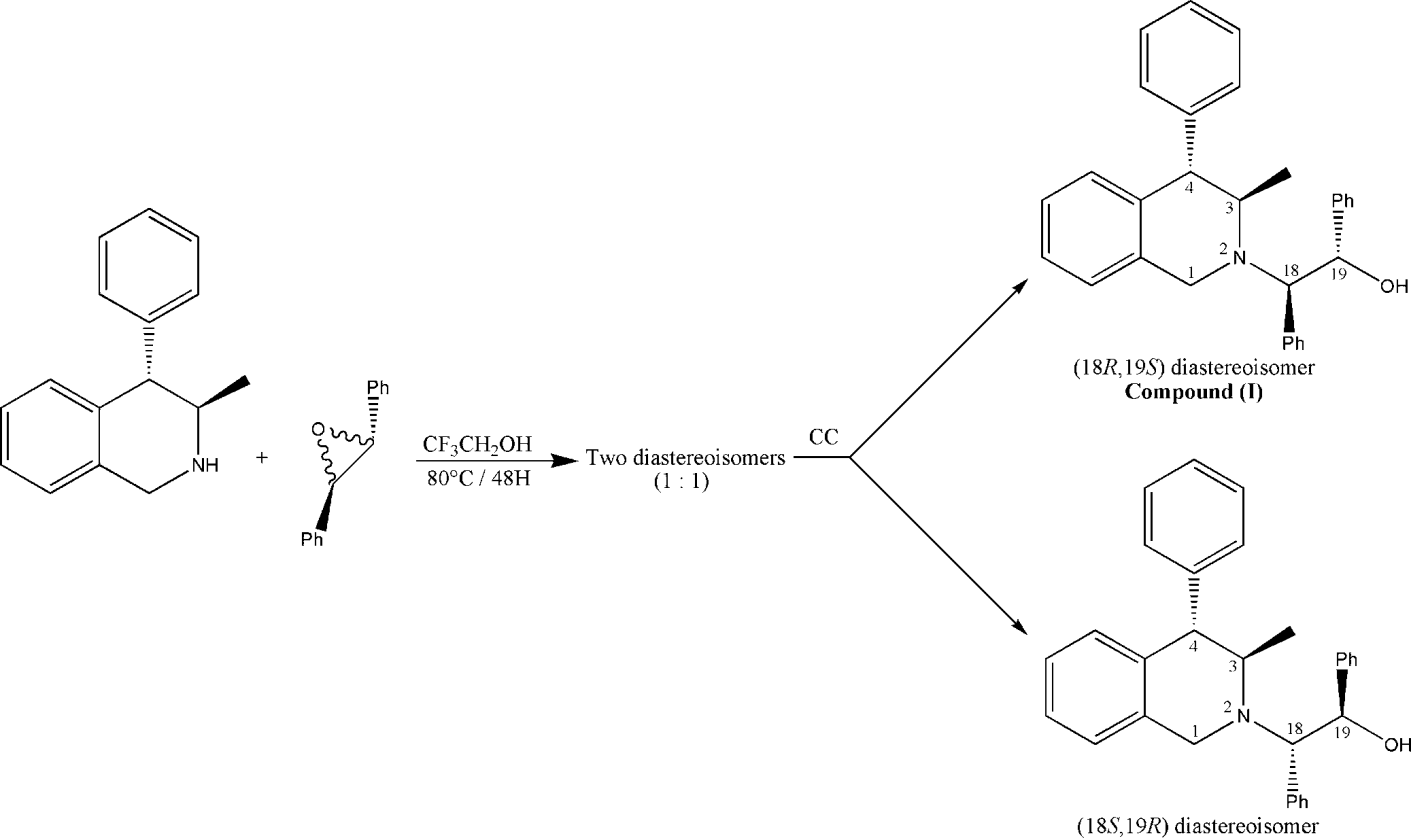


Among this family of chiral amino-alcohols is the title compound, (I), which we prepared through the alkyl­ation of tetra­hydro­iso­quinoline by the opening racemic *trans*-stilbene oxide reaction. Two diastereoisomers were obtained in a 1:1 ratio as determined by ^1^H NMR analysis on the crude mixture. These diastereoisomers were separated by column chromatography. The title mol­ecule was found to be the (*S,R*) diastereoisomer.

## Structural commentary   

**Figure 1:**
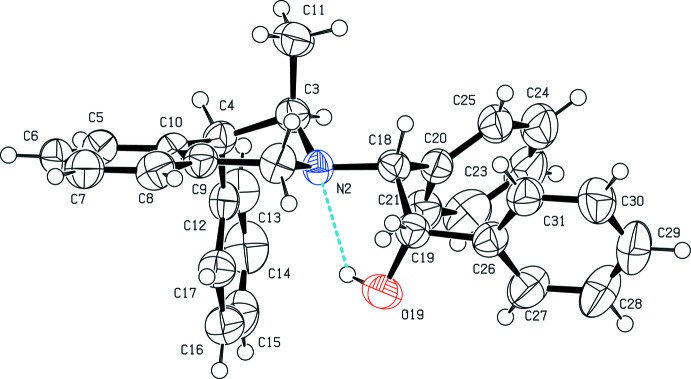
The mol­ecular structure of (I), showing the atom-labelling scheme. Displacement ellipsoids are drawn at the 50% probability level and H atoms are represented as small spheres of arbitrary radius. The dashed cyan line indicates the intra­molecular hydrogen bond between the hy­droxy group and the secondary amine.

The structure of (I) was confirmed using single crystal X-ray diffraction. The asymmetric unit of the ortho­rhom­bic unit cell comprises a single mol­ecule, shown in Fig. 1 ▸. The tetra­hydro­iso­quinoline unit is substituted by a methyl group in position 3, a phenyl substituent in position 4 and a β-alcohol substituent at the N atom. The heterocyclic ring exhibits a half-chair conformation, with atom C3 deviating by 0.706 (3) Å from the plane formed by atoms C1/N2/C4/C9/C10. The substituents in positions 3 and 4 of the heterocyclic ring are in axial positions. The mol­ecular structure of (I) is stabilized by an intra­molecular hydrogen bond between the hy­droxy O19—H19 group and atom N2, and to a lesser extent, between the aromatic C21—H21 and the phenyl group in position 4 (Table 1 ▸). By reference to two unchanging chiral C18 and C19 atoms, the mol­ecule was found to be the (18*R*,19*S*) diastereoisomer resulting from the reaction of tetra­hydro­iso­quinoline and the (*S*,*S*) *trans*-stilbene oxide enanti­omer.

**Figure 2:**
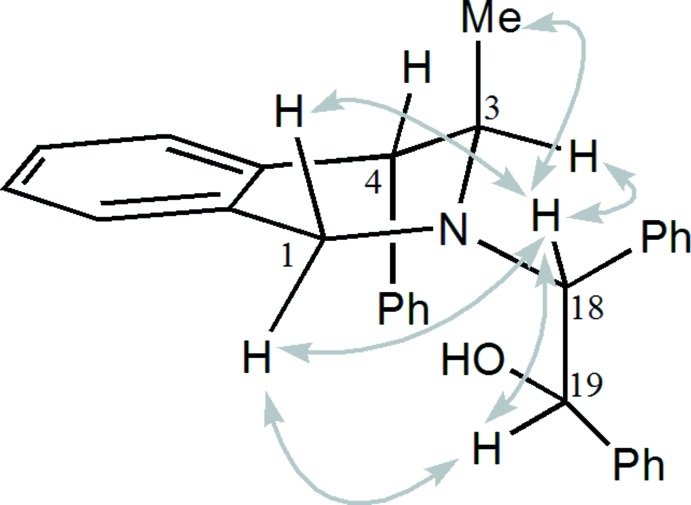
Selected NOESY correlations observed for compound (I).

This structure was confirmed through the means of usual 1D and 2D NMR experiments. NMR data show that the *trans* diequatorial arrangement of H3 and H4 is suggested by the coupling constant between H3 and H4 in ^1^H NMR (*J*_3,4_ ∼0 Hz), so the substituents C3-methyl and C4-phenyl are in an axial disposition. The absolute configurations of carbon atoms C18 and C19 were deduced from the NOESY maps to be *R* and *S*, respectively (Fig. 2 ▸).

## Supra­molecular features   

**Figure 3:**
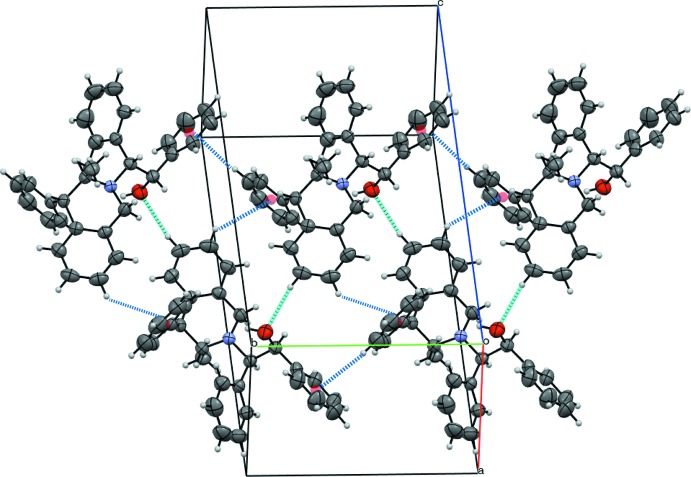
The ribbon structure of (I) formed along the *b*-axis direction *via* C—H⋯O inter­actions (cyan dashed lines) and C—H⋯π inter­actions (blue dashed lines). The red spheres indicate the centroids of the phenyl rings.

**Figure 4:**
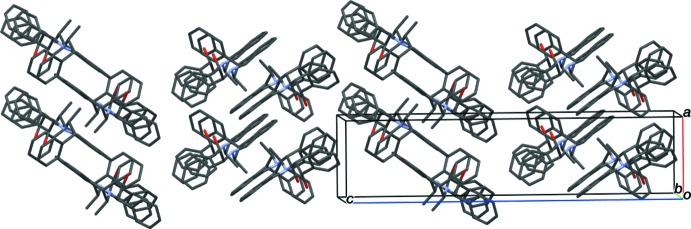
Crystal packing of compound (I) viewed down the *b*-axis direction. Ribbons stack in a herringbone arrangement with the phenyl groups at the column inter­face.

In the crystal, mol­ecules of (I) pack with no classical hydrogen bonds: the potential donor hydroxyl group is involved in an intra­molecular inter­action with the N atom. However, the oxygen atom acts as an acceptor in the short contact C6—H6⋯O19 (−*x*, 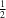 + *y*, 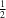 − *z*) with an O19⋯H distance of 2.57 Å, which is of the same order of magnitude of the H⋯O van der Waals distance (2.60 Å), whereas C—H⋯O contacts are frequently reported with H⋯O separations shorter than 2.4 Å (Taylor & Kennard, 1982 ▸). The N atom does not play a role in the packing as it is buried inside the structure. Nevertheless, these directed C—H⋯O inter­actions make an important contribution to the packing: zigzagging along the [010] direction, they pair mol­ecules in ribbons, placing the iso­quinoline moieties parallel to the (103) plane on both sides but without overlapping. The ribbon cohesion is reinforced by C—H⋯π inter­actions involving the phenyl group in position 4 and those attached to the β-alcohol part and which flank the ribbon, as shown in Fig. 3 ▸. They stack in the [100] direction as columns arranged in a herringbone manner but avoiding π-π- stacking (Fig. 4 ▸).

## Database survey   

A search of the Cambridge Structural Database, CSD (Version 5.40; *ConQuest* 1.21; Groom *et al.*, 2016 ▸) found 495 structures of tetra­hydro­iso­quinoline derivatives. Limiting the search to compounds with tri-substitutions on positions C3, C4 and the secondary amine N reduces the number of structures to seven: ADAGOC (Gzella *et al.*, 2006 ▸), JIPKEZ (White *et al.*, 2007 ▸), TIBPIE (Ben Ali *et al.*, 2007 ▸), VAHJOG (Davies *et al.*, 2016 ▸), XOSDUE (Gzella *et al.*, 2002 ▸), YEKKIK (Shi *et al.*, 2012 ▸) and ZIFSUE (Guo *et al.*, 2013 ▸). Except for the racemic VAHJOG, they all crystallize in the same *P*2_1_2_1_2_1_ space group. The structures of ZIFSUE, TIBPIE, VAHJOG, JIPKEZ and (I) superimpose well over the heterobicycle with the same conformation, unlike ADAGOC and XOSDUE which have a different half-chair configuration. The amino alcohol TIBPIE is obviously the closest related structure, differing in the N substitution of a cyclo­hexane carrying the hydroxyl group which is involved in the intra­molecular hydrogen bond.

## Hirshfeld surface analysis   

**Figure 5:**
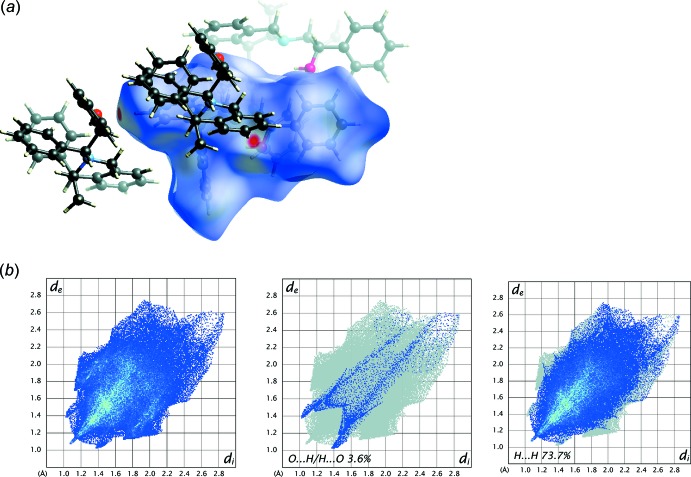
(*a*) View of the three-dimensional Hirshfeld surface mapped over *d*_norm_, over the range −0.1345 and +1.8231 arbitrary units, (*b*) the full two-dimensional fingerprint plot for (I) and the two-dimensional fingerprint plots for the O⋯H/H⋯O inter­actions and the H⋯H inter­actions

**Figure 6:**
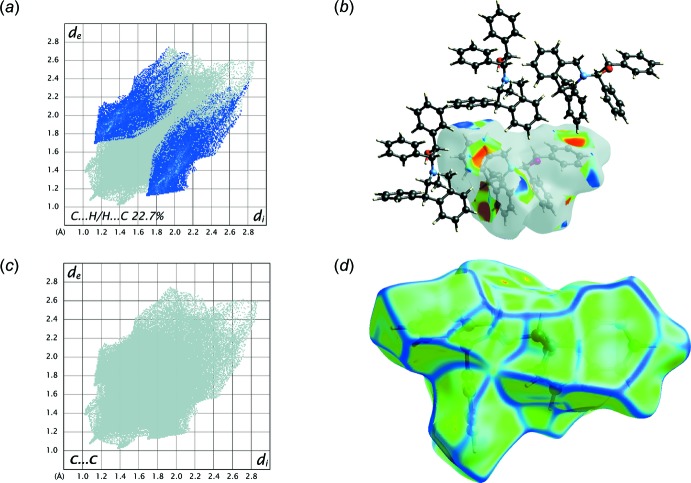
(*a*) The Hirshfeld surface mapped over the shape-index property, (*b*) the two-dimensional fingerprint plot for the H⋯C/C⋯H inter­actions, (*c*) the Hirshfeld surface mapped over curvedness and (*d*) the two-dimensional fingerprint plot for the C⋯C inter­actions in the title compound.

The inter­molecular inter­actions were qu­anti­fied using Hirshfeld surface analysis and the associated two-dimensional fingerprint plots using *CrystalExplorer17.5* (Turner *et al.*, 2017 ▸). The electrostatic potentials were calculated using *TONTO*, integrated within *CrystalExplorer*. The analysis of inter­molecular inter­actions through the mapping of *d*_norm_ presented in Fig. 5 ▸ compares the contact distances *d*_i_ and *d*_e_ from the Hirshfeld surface to the nearest atom inside and outside, respectively, with their respective van der Waals radii. The blue, white and red colour conventions recognize the inter­atomic contacts as longer, at van der Waals separations and short inter­atomic contacts. The C—H⋯O contacts are identified in the *d*_norm_-mapped surface as two red spots showing the inter­action between the neighbouring mol­ecules (Fig. 5 ▸ *a*). The overall two-dimensional fingerprint plot derived form the Hirshfeld surface is a useful method to summarize the frequency of each combination of *d*_e_ and *d*_i_ across the surface of the studied mol­ecule, encompassing all inter­molecular contacts (Fig. 5 ▸ *b*). The delineated fingerprint plots (Fig. 5 ▸ *b* and 6*a*,c) focus on specific inter­actions, providing information about the major and minor percentage contribution of inter­atomic contacts in the compound. The H⋯H inter­actions account for the three quarters of the total (73.7%) with an evident sting at about *d*_i_ = *d*_e_ = 1.1 Å (Fig. 5 ▸ *b*). The C⋯H/H⋯C plot, which refers to the C—H⋯π inter­actions previously described (22.7%,) shows two broad symmetrical wings at about *d*_i_ + *d*_e_ = 2.8 Å (Fig. 6 ▸ *a*). These inter­actions are observed as red regions on the shape-index surface (Fig. 6 ▸ *b*). The absence of C⋯C contacts, highlighted by the Hirshfeld surface with high curvedness delineated by dark-blue edges, confirms that no π–π stacking inter­actions take place in the crystal packing (Fig. 6 ▸ *c*,*d*). The third marginal contribution is O⋯H/H⋯O (3.6%) with a pair of sharp spikes at about *d*_i_ + *d*_e_ = 2.4 Å, symmetrically disposed with respect to the diagonal, indicating the presence of inter­molecular C—H⋯O inter­actions, which play a role in ordering the mol­ecules inside the crystal.

## Synthesis and crystallization   

The title *β*-amino alcohol was obtained by mixing racemic *trans*-stilbene oxide (5.1g, 26mmol) with (3*R*,4*S*)-3-methyl-4-phenyl-1,2,3,4-tetra­hydro­isoquinoleine (3g, 13mmol), which was prepared according to the method of Bohé *et al.* (1999 ▸).

The mixture was heated at 353.15 K for 48 h in CF_3_CH_2_OH (65 ml), the reaction being monitored by TLC. Two diastereoisomers were obtained in a 1:1 ratio. These diastereoisomers were separated by column chromatography. Only the title compound (white solid) was successfully recrystallized. Crystals were grown by placing this dastereoisomer in a minimum amount of hot heptane. [α] _D_^25^ = −23.6 (*c* 1, CHCl_3_), m.p. 425 K.

## Refinement   

Crystal data, data collection and structure refinement details are summarized in Table 2 ▸. H atoms were placed in calculated positions (C—H = 0.93–0.98 Å) and refined as riding with *U*_iso_(H) = 1.2*U*_eq_(C). The crystal studied was refined as a two-component inversion twin.

## Supplementary Material

Crystal structure: contains datablock(s) I. DOI: 10.1107/S2056989019011964/ff2162sup1.cif

Structure factors: contains datablock(s) I. DOI: 10.1107/S2056989019011964/ff2162Isup2.hkl

Supporting information file. DOI: 10.1107/S2056989019011964/ff2162Isup3.cml

CCDC reference: 1950166

Additional supporting information:  crystallographic information; 3D view; checkCIF report

## Figures and Tables

**Table 1:** Hydrogen-bond geometry (Å, °) *Cg*2, *Cg*3, *Cg*4 and *Cg*5 are the centroids of the C5–C10, C12-C17, C20–C25, and C26–C31 rings, respectively.

*D*—H⋯*A*	*D*—H	H⋯*A*	*D*⋯*A*	*D*—H⋯*A*
O19—H*OH*⋯N2	0.86 (3)	2.18 (3)	2.737 (2)	123 (2)
C27—H27⋯O19	0.93	2.48	2.798 (3)	100
C21—H21⋯*Cg*3	0.93	3.14	3.930 (4)	144
C6—H6⋯O19^i^	0.93	2.57	3.492 (3)	170
C14—H14⋯*Cg*5^ii^	0.93	2.95	3.770 (4)	147
C16—H16⋯*Cg*4^iii^	0.93	2.92	3.743 (3)	148
C31—H31⋯*Cg*2^iv^	0.93	2.96	3.803 (3)	152

Symmetry codes: (i) 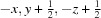; (ii) 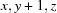; (iii) 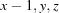; (iv) 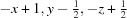.

**Table 2:** Experimental details

Crystal data	
Chemical formula	C_30_H_29_NO
*M*_r_	419.54
Crystal system, space group	Orthorhombic, *P*2_1_2_1_2_1_
Temperature (K)	293
*a*, *b*, *c* (Å)	7.3009 (8), 11.0552 (11), 30.006 (3)
*V* (Å^3^)	2421.8 (4)
*Z*	4
Radiation type	Mo *K*α
μ (mm^−1^)	0.07
Crystal size (mm)	0.59 × 0.45 × 0.35

Data collection	
Diffractometer	Nonius KappaCCD area detector
Absorption correction	Multi-scan (*CrysAlis PRO*; Rigaku OD, 2019 ▸)
*T*_min_, *T*_max_	0.844, 1.000
No. of measured, independent and observed [*I* > 2σ(*I*)] reflections	21751, 4427, 3948
*R*_int_	0.027
(sin θ/λ)_max_ (Å^−1^)	0.602

Refinement	
*R*[*F* ^2^ > 2σ(*F* ^2^)], *wR*(*F* ^2^), *S*	0.038, 0.083, 1.07
No. of reflections	4425
No. of parameters	295
H-atom treatment	H atoms treated by a mixture of independent and constrained refinement
Δρ_max_, Δρ_min_ (e Å^−3^)	0.11, −0.11
Absolute structure	Refined as an inversion twin.

Computer programs: *DENZO* (Otwinowski & Minor, 1997 ▸); *COLLECT* (Hooft, 1998 ▸), *CrysAlis PRO* (Rigaku OD, 2019 ▸), *SHELXT2014/5* (Sheldrick, 2015*a* ▸), *SHELXL2018/3* (Sheldrick, 2015*b* ▸).

## supplementary crystallographic information

### Crystal data

**Table:**

C_30_H_29_NO	*D*_x_ = 1.151 Mg m^−^^3^
*M**_r_* = 419.54	Mo *K*α radiation, λ = 0.71073 Å
Orthorhombic, *P*2_1_2_1_2_1_	Cell parameters from 6302 reflections
*a* = 7.3009 (8) Å	θ = 2.0–24.2°
*b* = 11.0552 (11) Å	µ = 0.07 mm^−^^1^
*c* = 30.006 (3) Å	*T* = 293 K
*V* = 2421.8 (4) Å^3^	Prism, colorless
*Z* = 4	0.59 × 0.45 × 0.35 mm
*F*(000) = 896	

### Data collection

**Table:**

Nonius KappaCCD area detector diffractometer	4427 independent reflections
Radiation source: 1.5kW sealed tube	3948 reflections with *I* > 2σ(*I*)
Graphite monochromator	*R*_int_ = 0.027
ω and φ scans	θ_max_ = 25.4°, θ_min_ = 2.7°
Absorption correction: multi-scan (CrysAlisPro; Rigaku OD, 2019)	*h* = −8→8
*T*_min_ = 0.844, *T*_max_ = 1.000	*k* = −13→13
21751 measured reflections	*l* = −36→36

### Refinement

**Table:**

Refinement on *F*^2^	Hydrogen site location: mixed
Least-squares matrix: full	H atoms treated by a mixture of independent and constrained refinement
*R*[*F*^2^ > 2σ(*F*^2^)] = 0.038	*w* = 1/[σ^2^(*F*_o_^2^) + (0.0304*P*)^2^ + 0.3321*P*] where *P* = (*F*_o_^2^ + 2*F*_c_^2^)/3
*wR*(*F*^2^) = 0.083	(Δ/σ)_max_ < 0.001
*S* = 1.07	Δρ_max_ = 0.11 e Å^−^^3^
4425 reflections	Δρ_min_ = −0.11 e Å^−^^3^
295 parameters	Extinction correction: SHELXL-2018/3 (Sheldrick 2015b), Fc^*^=kFc[1+0.001xFc^2^λ^3^/sin(2θ)]^-1/4^
0 restraints	Extinction coefficient: 0.0139 (11)
Primary atom site location: dual	Absolute structure: Refined as an inversion twin.
Secondary atom site location: difference Fourier map	

### Special details

**Table:**

Geometry. All esds (except the esd in the dihedral angle between two l.s. planes) are estimated using the full covariance matrix. The cell esds are taken into account individually in the estimation of esds in distances, angles and torsion angles; correlations between esds in cell parameters are only used when they are defined by crystal symmetry. An approximate (isotropic) treatment of cell esds is used for estimating esds involving l.s. planes.
Refinement. Refined as a 2-component inversion twin.

### Fractional atomic coordinates and isotropic or equivalent isotropic displacement parameters (Å^2^)

**Table:**

	*x*	*y*	*z*	*U*_iso_*/*U*_eq_	
C1	0.3286 (4)	−0.0015 (2)	0.21766 (7)	0.0459 (6)	
H1A	0.428447	−0.038350	0.234113	0.053*	
H1B	0.243483	−0.065133	0.209524	0.053*	
N2	0.4019 (2)	0.05352 (14)	0.17689 (6)	0.0355 (4)	
C3	0.5027 (3)	0.16552 (19)	0.18741 (7)	0.0400 (5)	
H3	0.556815	0.195001	0.159563	0.046*	
C4	0.3616 (3)	0.26023 (18)	0.20271 (7)	0.0379 (5)	
H4	0.430488	0.329448	0.214349	0.044*	
C5	0.1547 (3)	0.2891 (2)	0.26931 (7)	0.0471 (6)	
H5	0.162708	0.372208	0.264993	0.054*	
C6	0.0507 (3)	0.2447 (3)	0.30407 (8)	0.0553 (7)	
H6	−0.009196	0.297836	0.323159	0.064*	
C7	0.0357 (4)	0.1222 (3)	0.31050 (8)	0.0576 (7)	
H7	−0.035474	0.092057	0.333677	0.066*	
C8	0.1265 (3)	0.0443 (2)	0.28246 (8)	0.0518 (6)	
H8	0.116921	−0.038604	0.287084	0.060*	
C9	0.2328 (3)	0.0871 (2)	0.24723 (7)	0.0405 (5)	
C10	0.2479 (3)	0.2114 (2)	0.24055 (7)	0.0383 (5)	
C11	0.6589 (3)	0.1502 (2)	0.22101 (9)	0.0567 (7)	
H11A	0.726198	0.224575	0.223262	0.068*	
H11B	0.739191	0.086877	0.211187	0.068*	
H11C	0.609065	0.129700	0.249634	0.068*	
C12	0.2492 (3)	0.30571 (18)	0.16372 (7)	0.0400 (5)	
C13	0.3195 (4)	0.3956 (2)	0.13673 (8)	0.0570 (7)	
H13	0.431991	0.430129	0.143737	0.066*	
C14	0.2254 (5)	0.4350 (3)	0.09946 (9)	0.0707 (9)	
H14	0.275393	0.495164	0.081543	0.081*	
C15	0.0593 (5)	0.3858 (3)	0.08888 (9)	0.0699 (9)	
H15	−0.003927	0.412441	0.063832	0.080*	
C16	−0.0138 (4)	0.2967 (3)	0.11542 (9)	0.0623 (7)	
H16	−0.126869	0.262995	0.108399	0.072*	
C17	0.0809 (3)	0.2573 (2)	0.15257 (8)	0.0488 (6)	
H17	0.030362	0.197119	0.170385	0.056*	
C18	0.5032 (3)	−0.03477 (18)	0.14947 (7)	0.0367 (5)	
H18	0.600714	−0.070012	0.167776	0.042*	
C19	0.3684 (3)	−0.13740 (19)	0.13610 (8)	0.0416 (5)	
H19	0.338016	−0.182861	0.163143	0.048*	
O19	0.2046 (2)	−0.08765 (16)	0.11933 (6)	0.0560 (5)	
HOH	0.198 (4)	−0.016 (3)	0.1307 (9)	0.067*	
C20	0.5896 (3)	0.02111 (19)	0.10855 (7)	0.0394 (5)	
C21	0.4942 (4)	0.0997 (2)	0.08094 (8)	0.0553 (7)	
H21	0.377223	0.125320	0.088719	0.064*	
C22	0.5742 (5)	0.1398 (3)	0.04165 (9)	0.0730 (8)	
H22	0.510686	0.192712	0.023154	0.084*	
C23	0.7467 (5)	0.1018 (3)	0.02999 (10)	0.0778 (10)	
H23	0.798359	0.127746	0.003312	0.089*	
C24	0.8428 (4)	0.0263 (3)	0.05729 (10)	0.0685 (8)	
H24	0.960600	0.001927	0.049589	0.079*	
C25	0.7644 (3)	−0.0137 (2)	0.09627 (8)	0.0502 (6)	
H25	0.830335	−0.065258	0.114775	0.058*	
C26	0.4523 (3)	−0.22470 (19)	0.10333 (7)	0.0434 (5)	
C27	0.4150 (4)	−0.2197 (2)	0.05843 (9)	0.0648 (8)	
H27	0.330932	−0.163529	0.047712	0.075*	
C28	0.5013 (5)	−0.2974 (3)	0.02915 (9)	0.0816 (10)	
H28	0.474735	−0.293390	−0.001130	0.094*	
C29	0.6252 (5)	−0.3798 (3)	0.04431 (11)	0.0786 (9)	
H29	0.684240	−0.431168	0.024431	0.090*	
C30	0.6622 (4)	−0.3865 (2)	0.08887 (11)	0.0693 (8)	
H30	0.746120	−0.442975	0.099374	0.080*	
C31	0.5752 (4)	−0.3097 (2)	0.11835 (9)	0.0533 (6)	
H31	0.599963	−0.315488	0.148673	0.061*	

### Atomic displacement parameters (Å^2^)

**Table:**

	*U*^11^	*U*^22^	*U*^33^	*U*^12^	*U*^13^	*U*^23^
C1	0.0562 (15)	0.0388 (12)	0.0426 (13)	−0.0048 (11)	0.0068 (11)	0.0038 (10)
N2	0.0361 (10)	0.0336 (8)	0.0369 (9)	−0.0008 (8)	0.0058 (8)	0.0001 (7)
C3	0.0384 (12)	0.0391 (11)	0.0426 (12)	−0.0037 (10)	0.0039 (10)	−0.0003 (9)
C4	0.0419 (13)	0.0322 (10)	0.0395 (12)	−0.0059 (10)	0.0006 (10)	−0.0037 (9)
C5	0.0441 (13)	0.0536 (14)	0.0437 (13)	0.0038 (11)	−0.0043 (11)	−0.0078 (11)
C6	0.0421 (14)	0.0826 (19)	0.0413 (13)	0.0074 (14)	0.0005 (11)	−0.0127 (13)
C7	0.0482 (15)	0.0850 (19)	0.0395 (13)	−0.0076 (14)	0.0064 (12)	0.0015 (13)
C8	0.0552 (15)	0.0596 (14)	0.0406 (13)	−0.0126 (13)	0.0031 (12)	0.0036 (11)
C9	0.0423 (13)	0.0472 (12)	0.0320 (11)	−0.0030 (11)	0.0010 (10)	0.0002 (9)
C10	0.0364 (12)	0.0442 (12)	0.0342 (11)	0.0002 (10)	−0.0019 (10)	−0.0030 (9)
C11	0.0433 (14)	0.0589 (15)	0.0680 (17)	−0.0014 (12)	−0.0081 (13)	−0.0064 (13)
C12	0.0494 (14)	0.0319 (10)	0.0387 (12)	0.0043 (11)	0.0068 (10)	−0.0036 (9)
C13	0.0653 (17)	0.0470 (13)	0.0586 (16)	−0.0024 (13)	0.0074 (14)	0.0085 (12)
C14	0.093 (2)	0.0637 (17)	0.0558 (17)	0.0100 (18)	0.0123 (17)	0.0203 (14)
C15	0.091 (2)	0.0766 (19)	0.0426 (15)	0.0331 (19)	−0.0020 (15)	0.0037 (14)
C16	0.0589 (16)	0.0737 (17)	0.0544 (16)	0.0111 (15)	−0.0078 (14)	−0.0108 (14)
C17	0.0526 (15)	0.0485 (13)	0.0452 (13)	0.0024 (12)	−0.0009 (11)	0.0013 (11)
C18	0.0336 (11)	0.0391 (11)	0.0375 (11)	0.0034 (10)	−0.0016 (10)	0.0010 (9)
C19	0.0396 (12)	0.0390 (11)	0.0463 (13)	0.0003 (10)	−0.0006 (10)	−0.0009 (10)
O19	0.0414 (9)	0.0551 (10)	0.0715 (12)	0.0037 (8)	−0.0092 (9)	−0.0069 (9)
C20	0.0415 (13)	0.0403 (11)	0.0364 (12)	−0.0010 (10)	0.0015 (10)	−0.0042 (9)
C21	0.0617 (17)	0.0595 (14)	0.0448 (14)	0.0047 (14)	0.0015 (12)	0.0049 (12)
C22	0.097 (2)	0.0752 (19)	0.0467 (15)	−0.0039 (19)	−0.0026 (17)	0.0129 (14)
C23	0.095 (3)	0.091 (2)	0.0469 (16)	−0.024 (2)	0.0244 (17)	−0.0042 (16)
C24	0.0622 (18)	0.082 (2)	0.0616 (18)	−0.0138 (17)	0.0209 (15)	−0.0176 (16)
C25	0.0452 (14)	0.0532 (14)	0.0522 (15)	−0.0035 (12)	0.0073 (11)	−0.0090 (11)
C26	0.0456 (14)	0.0384 (11)	0.0461 (13)	−0.0055 (11)	−0.0012 (11)	−0.0046 (10)
C27	0.086 (2)	0.0577 (15)	0.0507 (16)	0.0103 (16)	−0.0131 (15)	−0.0060 (12)
C28	0.122 (3)	0.076 (2)	0.0477 (16)	0.007 (2)	−0.0009 (18)	−0.0158 (15)
C29	0.095 (2)	0.0622 (18)	0.078 (2)	0.0071 (19)	0.0216 (19)	−0.0239 (16)
C30	0.069 (2)	0.0568 (16)	0.082 (2)	0.0137 (15)	0.0011 (17)	−0.0151 (15)
C31	0.0581 (16)	0.0453 (12)	0.0563 (15)	0.0058 (12)	−0.0049 (13)	−0.0066 (11)

### Geometric parameters (Å, º)

**Table:**

C1—N2	1.467 (3)	C16—C17	1.382 (3)
C1—C9	1.496 (3)	C16—H16	0.9300
C1—H1A	0.9700	C17—H17	0.9300
C1—H1B	0.9700	C18—C20	1.512 (3)
N2—C3	1.475 (3)	C18—C19	1.555 (3)
N2—C18	1.475 (3)	C18—H18	0.9800
C3—C11	1.531 (3)	C19—O19	1.409 (3)
C3—C4	1.539 (3)	C19—C26	1.508 (3)
C3—H3	0.9800	C19—H19	0.9800
C4—C10	1.507 (3)	O19—HOH	0.86 (3)
C4—C12	1.515 (3)	C20—C25	1.383 (3)
C4—H4	0.9800	C20—C21	1.388 (3)
C5—C6	1.380 (3)	C21—C22	1.389 (4)
C5—C10	1.395 (3)	C21—H21	0.9300
C5—H5	0.9300	C22—C23	1.373 (4)
C6—C7	1.372 (4)	C22—H22	0.9300
C6—H6	0.9300	C23—C24	1.364 (4)
C7—C8	1.374 (4)	C23—H23	0.9300
C7—H7	0.9300	C24—C25	1.375 (4)
C8—C9	1.394 (3)	C24—H24	0.9300
C8—H8	0.9300	C25—H25	0.9300
C9—C10	1.393 (3)	C26—C31	1.375 (3)
C11—H11A	0.9600	C26—C27	1.376 (3)
C11—H11B	0.9600	C27—C28	1.381 (4)
C11—H11C	0.9600	C27—H27	0.9300
C12—C13	1.380 (3)	C28—C29	1.362 (4)
C12—C17	1.381 (3)	C28—H28	0.9300
C13—C14	1.383 (4)	C29—C30	1.366 (4)
C13—H13	0.9300	C29—H29	0.9300
C14—C15	1.367 (4)	C30—C31	1.381 (4)
C14—H14	0.9300	C30—H30	0.9300
C15—C16	1.374 (4)	C31—H31	0.9300
C15—H15	0.9300		
			
N2—C1—C9	113.18 (17)	C16—C15—H15	120.2
N2—C1—H1A	108.9	C15—C16—C17	119.9 (3)
C9—C1—H1A	108.9	C15—C16—H16	120.0
N2—C1—H1B	108.9	C17—C16—H16	120.0
C9—C1—H1B	108.9	C12—C17—C16	121.2 (2)
H1A—C1—H1B	107.8	C12—C17—H17	119.4
C1—N2—C3	110.59 (16)	C16—C17—H17	119.4
C1—N2—C18	111.93 (16)	N2—C18—C20	113.06 (16)
C3—N2—C18	115.13 (16)	N2—C18—C19	108.02 (16)
N2—C3—C11	114.83 (18)	C20—C18—C19	110.66 (17)
N2—C3—C4	107.52 (17)	N2—C18—H18	108.3
C11—C3—C4	112.15 (18)	C20—C18—H18	108.3
N2—C3—H3	107.3	C19—C18—H18	108.3
C11—C3—H3	107.3	O19—C19—C26	111.22 (19)
C4—C3—H3	107.3	O19—C19—C18	110.15 (17)
C10—C4—C12	113.71 (18)	C26—C19—C18	112.22 (18)
C10—C4—C3	110.49 (17)	O19—C19—H19	107.7
C12—C4—C3	110.99 (17)	C26—C19—H19	107.7
C10—C4—H4	107.1	C18—C19—H19	107.7
C12—C4—H4	107.1	C19—O19—HOH	105 (2)
C3—C4—H4	107.1	C25—C20—C21	118.6 (2)
C6—C5—C10	121.1 (2)	C25—C20—C18	119.2 (2)
C6—C5—H5	119.4	C21—C20—C18	122.1 (2)
C10—C5—H5	119.4	C20—C21—C22	119.7 (3)
C7—C6—C5	120.1 (2)	C20—C21—H21	120.1
C7—C6—H6	120.0	C22—C21—H21	120.1
C5—C6—H6	120.0	C23—C22—C21	120.3 (3)
C6—C7—C8	119.6 (2)	C23—C22—H22	119.9
C6—C7—H7	120.2	C21—C22—H22	119.9
C8—C7—H7	120.2	C24—C23—C22	120.4 (3)
C7—C8—C9	121.3 (2)	C24—C23—H23	119.8
C7—C8—H8	119.3	C22—C23—H23	119.8
C9—C8—H8	119.3	C23—C24—C25	119.5 (3)
C10—C9—C8	119.2 (2)	C23—C24—H24	120.2
C10—C9—C1	121.60 (19)	C25—C24—H24	120.2
C8—C9—C1	119.2 (2)	C24—C25—C20	121.4 (3)
C9—C10—C5	118.7 (2)	C24—C25—H25	119.3
C9—C10—C4	120.36 (19)	C20—C25—H25	119.3
C5—C10—C4	121.0 (2)	C31—C26—C27	118.6 (2)
C3—C11—H11A	109.5	C31—C26—C19	119.3 (2)
C3—C11—H11B	109.5	C27—C26—C19	122.2 (2)
H11A—C11—H11B	109.5	C26—C27—C28	120.5 (3)
C3—C11—H11C	109.5	C26—C27—H27	119.8
H11A—C11—H11C	109.5	C28—C27—H27	119.8
H11B—C11—H11C	109.5	C29—C28—C27	120.4 (3)
C13—C12—C17	117.9 (2)	C29—C28—H28	119.8
C13—C12—C4	119.3 (2)	C27—C28—H28	119.8
C17—C12—C4	122.7 (2)	C28—C29—C30	119.7 (3)
C12—C13—C14	121.1 (3)	C28—C29—H29	120.2
C12—C13—H13	119.5	C30—C29—H29	120.2
C14—C13—H13	119.5	C29—C30—C31	120.2 (3)
C15—C14—C13	120.2 (3)	C29—C30—H30	119.9
C15—C14—H14	119.9	C31—C30—H30	119.9
C13—C14—H14	119.9	C26—C31—C30	120.7 (3)
C14—C15—C16	119.7 (3)	C26—C31—H31	119.7
C14—C15—H15	120.2	C30—C31—H31	119.7
			
C9—C1—N2—C3	47.4 (2)	C13—C12—C17—C16	0.5 (3)
C9—C1—N2—C18	177.26 (19)	C4—C12—C17—C16	−176.6 (2)
C1—N2—C3—C11	56.3 (2)	C15—C16—C17—C12	−0.1 (4)
C18—N2—C3—C11	−71.7 (2)	C1—N2—C18—C20	−176.66 (18)
C1—N2—C3—C4	−69.2 (2)	C3—N2—C18—C20	−49.2 (2)
C18—N2—C3—C4	162.68 (16)	C1—N2—C18—C19	60.5 (2)
N2—C3—C4—C10	54.4 (2)	C3—N2—C18—C19	−172.04 (17)
C11—C3—C4—C10	−72.8 (2)	N2—C18—C19—O19	48.5 (2)
N2—C3—C4—C12	−72.7 (2)	C20—C18—C19—O19	−75.7 (2)
C11—C3—C4—C12	160.16 (19)	N2—C18—C19—C26	173.03 (17)
C10—C5—C6—C7	−0.8 (4)	C20—C18—C19—C26	48.8 (2)
C5—C6—C7—C8	0.8 (4)	N2—C18—C20—C25	138.8 (2)
C6—C7—C8—C9	−0.6 (4)	C19—C18—C20—C25	−99.9 (2)
C7—C8—C9—C10	0.4 (4)	N2—C18—C20—C21	−46.1 (3)
C7—C8—C9—C1	−179.9 (2)	C19—C18—C20—C21	75.2 (2)
N2—C1—C9—C10	−12.7 (3)	C25—C20—C21—C22	1.0 (4)
N2—C1—C9—C8	167.7 (2)	C18—C20—C21—C22	−174.2 (2)
C8—C9—C10—C5	−0.4 (3)	C20—C21—C22—C23	0.2 (4)
C1—C9—C10—C5	179.9 (2)	C21—C22—C23—C24	−1.4 (5)
C8—C9—C10—C4	−179.9 (2)	C22—C23—C24—C25	1.3 (4)
C1—C9—C10—C4	0.4 (3)	C23—C24—C25—C20	−0.1 (4)
C6—C5—C10—C9	0.6 (3)	C21—C20—C25—C24	−1.1 (3)
C6—C5—C10—C4	−179.8 (2)	C18—C20—C25—C24	174.2 (2)
C12—C4—C10—C9	104.1 (2)	O19—C19—C26—C31	−159.3 (2)
C3—C4—C10—C9	−21.5 (3)	C18—C19—C26—C31	76.8 (3)
C12—C4—C10—C5	−75.4 (2)	O19—C19—C26—C27	23.1 (3)
C3—C4—C10—C5	159.02 (19)	C18—C19—C26—C27	−100.8 (3)
C10—C4—C12—C13	152.5 (2)	C31—C26—C27—C28	−0.9 (4)
C3—C4—C12—C13	−82.2 (2)	C19—C26—C27—C28	176.8 (3)
C10—C4—C12—C17	−30.5 (3)	C26—C27—C28—C29	−0.2 (5)
C3—C4—C12—C17	94.8 (2)	C27—C28—C29—C30	0.9 (5)
C17—C12—C13—C14	−0.7 (4)	C28—C29—C30—C31	−0.4 (5)
C4—C12—C13—C14	176.5 (2)	C27—C26—C31—C30	1.4 (4)
C12—C13—C14—C15	0.5 (4)	C19—C26—C31—C30	−176.4 (2)
C13—C14—C15—C16	−0.1 (4)	C29—C30—C31—C26	−0.8 (4)
C14—C15—C16—C17	−0.1 (4)		

### Hydrogen-bond geometry (Å, º)

*Cg*2, *Cg*3, *Cg*4 and *Cg*5 are the centroids of the C5–C10, C12-C17, C20–C25, and C26–C31 rings, respectively.

**Table:**

*D*—H···*A*	*D*—H	H···*A*	*D*···*A*	*D*—H···*A*
O19—H*OH*···N2	0.86 (3)	2.18 (3)	2.737 (2)	123 (2)
C27—H27···O19	0.93	2.48	2.798 (3)	100
C21—H21···*Cg*3	0.93	3.14	3.930 (4)	144
C6—H6···O19^i^	0.93	2.57	3.492 (3)	170
C14—H14···*Cg*5^ii^	0.93	2.95	3.770 (4)	147
C16—H16···*Cg*4^iii^	0.93	2.92	3.743 (3)	148
C31—H31···*Cg*2^iv^	0.93	2.96	3.803 (3)	152

Symmetry codes: (i) −*x*, *y*+1/2, −*z*+1/2; (ii) *x*, *y*+1, *z*; (iii) *x*−1, *y*, *z*; (iv) −*x*+1, *y*−1/2, −*z*+1/2.
